# High Prevalence of *Mansonella ozzardi* Infection in the Amazon Region, Ecuador

**DOI:** 10.3201/eid2511.181964

**Published:** 2019-11

**Authors:** Manuel Calvopina, Carlos Chiluisa-Guacho, Alberto Toapanta, David Fonseca, Irina Villacres

**Affiliations:** Universidad de las Américas, Quito, Ecuador (M. Calvopina, A. Toapanta, D. Fonseca, I. Villacres);; Instituto Nacional de Investigación en Salud Pública, Tena, Ecuador (C. Chiluisa-Guacho)

**Keywords:** Mansonella ozzardi, mansonelliasis, parasites, infection, filariasis, nematodes, prevalence, endemicity, vector-borne infections, zoonoses, Amazon Region, Ecuador

## Abstract

We reviewed Giemsa-stained thick blood smears, obtained through the national malaria surveillance program in the Amazon region of Ecuador, by light microscopy for *Mansonella* spp. microfilariae. Of 2,756 slides examined, 566 (20.5%) were positive. Nested PCR confirmed that the microfilariae were those of *M. ozzardi* nematodes, indicating that this parasite is endemic to this region.

Although mansonelliases is probably the most prevalent filarial infection worldwide, it is the least studied and is considered a neglected parasite infection (*1**,**2*). Human infection with members of the filarial nematode genus *Mansonella*, including *M. ozzardi*, *M. perstans*, and *M. streptocerca* nematodes, is common and widespread in the Western Hemisphere and Africa. *M. ozzardi* nematodes are found exclusively in the Western Hemisphere from southern Mexico to northwestern Argentina but have not been reported in Ecuador, Chile, Uruguay, and Paraguay (*1**,**2*). *M. perstans* infections are found mainly in sub-Saharan Africa, with sporadic cases in a few countries in South America, whereas *M. streptocerca* infections are found only in Africa (*1**–**4*).

Epidemiologic studies have reported that *M. ozzardi* nematodes are highly prevalent in the Amazon Basin (Brazil, Colombia, Peru, Bolivia, Venezuela, and Argentina) and on some Caribbean islands. In the general population, the prevalence rate of infection ranges from 0% to 46%; however, in some areas it is 92.3% (*2*).

Community health workers from the malaria control program in the Amazon region of Ecuador have reported that filaria-like nematodes are seen in thick blood smears, suggesting the presence of a *Mansonella* spp. in this area. *Mansonella* sp. nematodes are transmitted by dipteran flies. In particular, *M. ozzardi* nematodes are transmitted by biting midges of the genera *Culicoides* and by black flies from the genus *Simulium* (*1*). Both of these vectors are present in the Amazon region of Ecuador (Renato Leon, Universidad San Francisco de Quito, pers. comm., 2018 Dec 5).

Endemic areas for malaria and *M. ozzardi* infection often overlap; thus, microfilariae might be found on thick blood smears prepared for malaria diagnosis (*2*). Microfilariae of *M. ozzardi* can be easily distinguished morphologically from those of *M. perstans* by examination of the tail end: unlike *M. ozzardi* nematodes, the tails of *M. perstans* nematodes are blunt and have nuclei extending to the tail end (*5*). PCR-based amplification of species-specific target sequences results in increased diagnostic sensitivity and reliable differentiation between *M. ozzardi* nematodes and co-endemic filarial species, such as *M. perstans* and *Onchocerca volvulus* nematodes (*6*). Therefore, we conducted a retrospective study to document presence of human infections with *Mansonella* spp. nematodes in the Amazon region of Ecuador. The study protocol was approved by the Ethics Committee of the Instituto Nacional de Investigación en Salud Pública (CEISH-INSPI-013).

## The Study

We conducted a study in 5 provinces (Sucumbíos, Orellana, Napo, Pastaza, and Morona Santiago) in the Amazon rainforest region of Ecuador for which malaria slides were available. The Amazon region of Ecuador covers ≈40% of the area of this country, extends from the eastern Andes to the lowlands of the Amazon basin, and borders Colombia and Peru.

The study population was composed of mestizos and Kichwa, Shuar, and Achuar indigenous groups. Community health workers collected blood samples by finger prick from 7:00 am until 7:00 pm from persons suspected of having malaria. Malaria centers are located throughout the rainforest in an ongoing program for malaria control under the guidance of the Ministry of Public Health.

Thick and thin blood smears were stained with Giemsa and viewed by microscopy at 100× magnification under oil immersion for *Plasmodium* spp. parasites. We retrospectively screened all slides obtained during 2014–2015 for *Mansonella* spp. nematodes.

Although >5,000 stained blood smears were reviewed, only 2,756 slides could be read because of poor preservation. We examined thick and thin blood smears by using light microscopy and a 20× objective lens to detect microfilariae and a 40× objective lens for species identification. No epidemiologic data were available because we conducted a retrospective analysis.

**Figure 1:**
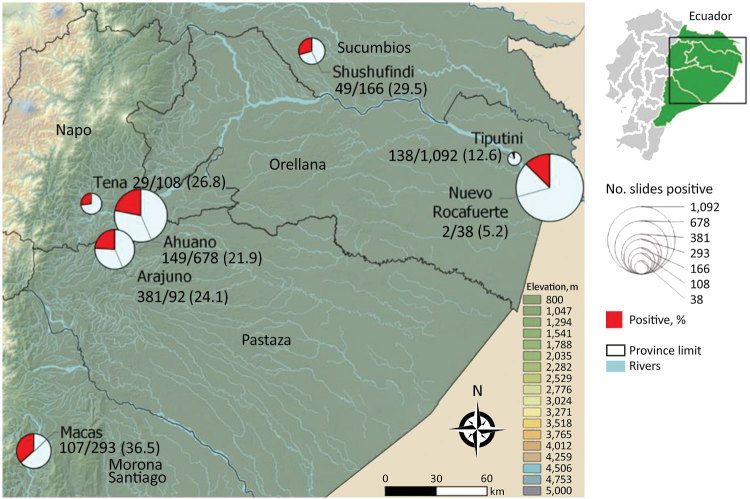
Amazon region of Ecuador where testing for *Mansonella ozzardi* microfilariae in humans was conducted. Of 2,756 archived slides from human infections, 566 (20.5%) were positive for this parasite. Values are no. positive/no. tested (%). Inset shows location of study area within Ecuador.

Of 2,756 Giemsa-stained blood smears examined, we detected microfilariae of *Mansonella* spp. nematodes in 566 (20.5%). Microfilariae were unsheathed (average length 155 µm–212 µm) and had tapered, nonnucleated tails; anterior extremities ended in cephalic spaces. On the basis of these morphologic characteristics, we identified all filarial infections as *M. ozzardi* nematodes. Many microfilariae appeared damaged and partially destroyed. No microfilariae with the characteristics of *M. perstans* nematodes were observed. Infection rates between provinces ranged from 5.2% to 36.5%; infections were most prevalent in Morona Santiago, which borders Peru (Figure 1).

**Figure 2:**
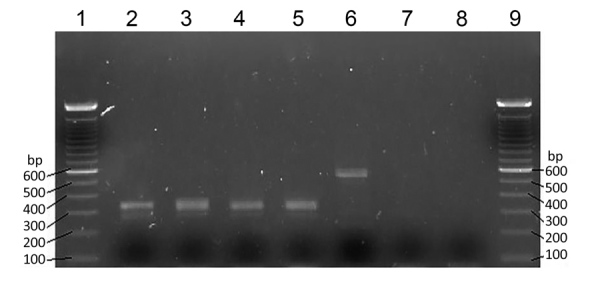
Nested PCR amplification products for *Mansonella ozzardi* microfilariae obtained from archived human samples in the Amazon region of Ecuador. Samples were subjected to electrophoresis on a 2% agarose gel. Lanes 1 and 9, 100-bp molecular mass ladders; lanes 2, 3, 4, and 5, *M. ozzardi* nematode-positive samples (sample nos. 14, 53, 27, and 25, respectively) that yielded a 305-bp fragment; lane 6, *Toxocara canis* roundworm (610-bp fragment); lane 7, *M. ozzardi* nematode–negative thick blood smear; lane 8, negative control. These PCR results confirmed data obtained by microscopy. Morphologic characteristics of and DNA findings for the microfilariae indicated that this parasite was an *M. ozzardi* nematode.

We then extracted DNA from all microscope slides positive for microfilariae. We scraped blood films off the slides into 70% ethanol. After microcentrifugation at 8,000 rpm for 2 min, we subjected supernatants to DNA extraction by using Chelex treatment (*7*), followed by proteinase K digestion. We performed a nested PCR according to the method of Tang et al. (*6*). This PCR amplifies the internal transcribed spacer region 1 of the rDNA gene of filarial species. The size of this region varies among *O. volvulus*, *M. ozzardi*, and *M. perstans* nematodes, and the PCR yields amplicons of different sizes for each species (*6*). Expected product sizes were 305 bp for *M. ozzardi* nematodes and 312 bp for *M. perstans* nematodes. PCR products after the second amplification were subjected to electrophoresis on 2% agarose gels (Figure 2).

## Conclusions

We report a high frequency of autochthonous human mansonelliasis caused by *M. ozzardi* nematodes in the Amazon region of Ecuador. These parasites were identified by microscope identification of characteristic microfilariae and detection of species-specific DNA.

This study showed that human infections with *M. ozzardi* nematodes are highly prevalent throughout the Amazon region of Ecuador. High rates of circulating microfilariae are strongly suggestive of active local transmission, particularly because known vectors (biting midges of the genus *Culicoides* and black flies of the genus *Simulium*) are present in the surveyed region. Further entomologic studies are needed to identify specific Diptera species involved in local transmission of *M. ozzardi* nematodes in Ecuador.

Prevalence of *M. ozzardi* infection ranged from 5.2% to 36.5% in this study, similar to infection rates reported in bordering regions of Colombia and Peru, indicating that this region is probably a large focus of infection extending throughout several countries in the Amazon region (*2*). *M. ozzardi* infection rates were higher than those reported from Brazil, but considerably lower than those for the indigenous population of El Vaupés in Colombia, where the infection rate was 96% (*8*). However, in estimating prevalence by using stored, partially degraded blood smears and light microscopy, we might have underestimated the true prevalence. Use of FTA cards (Whatman, https://www.sigmaaldrich.com) has been demonstrated to be more sensitive for detection of *M. ozzardi* nematodes (*9*).

The morphologic features of microfilariae we observed were typical for *M. ozzardi* nematodes, and nested PCR confirmed detection of a DNA fragment (305 bp) known to be specific for this parasite. This assay used universal filariae PCR primers to amplify a variable portion of filarial parasite internal transcribed spacer 1 rDNA gene and enabled subsequent differentiation of species on the basis of the size of the amplified fragment (*6*). In addition, DNA was successfully extracted from scrapings of thick and thin dried blood films, even after long storage periods of 2–3 years.

We observed no microfilariae of *M. perstans* nematodes, and no positive samples showed DNA fragment sizes representative of this nematode (*6*). *M. perstans* nematodes have been reported only in the northern part of the Amazon rainforest from equatorial Brazil to the Caribbean coast of South America (*1*). In Colombia, *M. perstans* nematodes have been observed in a restricted area among the Curripacos Amerindians in the Comisaría del Guainia region bordering Venezuela (*10*), but have not been found in the Peruvian Amazon (*1**,**2*). However, further prospective studies using molecular techniques, are needed to clarify the epidemiologic status of this parasite.

In summary, we retrospectively identified human infection with *M. ozzardi* nematodes in the Amazon region of Ecuador. Our findings confirm that this parasite is endemic to this region.
